# Lasofoxifene as a potential treatment for aromatase inhibitor-resistant ER-positive breast cancer

**DOI:** 10.1186/s13058-024-01843-4

**Published:** 2024-06-07

**Authors:** Muriel Lainé, Marianne E Greene, Justyna D Kurleto, Grazyna Bozek, Tiffany Leng, Rosemary J Huggins, Barry S Komm, Geoffrey L Greene

**Affiliations:** 1 The Ben May Department for Cancer Research, The University of Chicago, 929 East 57th Street GCIS W421C, Chicago, IL 60637 USA; 2 Sermonix Pharmaceuticals, Columbus, OH USA

**Keywords:** Breast cancer, Endocrine resistant, Estrogen receptor, Fulvestrant, HER2-positive, Lasofoxifene, Letrozole, Selective estrogen receptor modulator

## Abstract

**Background:**

Breast cancers treated with aromatase inhibitors (AIs) can develop AI resistance, which is often driven by estrogen receptor-alpha (ERα/*ESR1*) activating mutations, as well as by ER-independent signaling pathways. The breast ER antagonist lasofoxifene, alone or combined with palbociclib, elicited antitumor activities in a xenograft model of ER + metastatic breast cancer (mBC) harboring *ESR1* mutations. The current study investigated the activity of LAS in a letrozole-resistant breast tumor model that does not have *ESR1* mutations.

**Methods:**

Letrozole-resistant, MCF7 LTLT cells tagged with luciferase-GFP were injected into the mammary duct inguinal glands of NSG mice (MIND model; 6 mice/group). Mice were randomized to vehicle, lasofoxifene ± palbociclib, fulvestrant ± palbociclib, or palbociclib alone 2–3 weeks after cell injections. Tumor growth and metastases were monitored with in vivo and ex vivo luminescence imaging, terminal tumor weight measurements, and histological analysis. The experiment was repeated with the same design and 8–9 mice in each treatment group.

**Results:**

Western blot analysis showed that the MCF7 LTLT cells had lower ERα and higher HER2 expressions compared with normal MCF7 cells. Lasofoxifene ± palbociclib, but not fulvestrant, significantly reduced primary tumor growth versus vehicle as assessed by in vivo imaging of tumors at study ends. Percent tumor area in excised mammary glands was significantly lower for lasofoxifene plus palbociclib versus vehicle. Ki67 staining showed decreased overall tumor cell proliferation with lasofoxifene ± palbociclib. The lasofoxifene + palbociclib combination was also associated with significantly fewer bone metastases compared with vehicle. Similar results were observed in the repeat experiment.

**Conclusions:**

In a mouse model of letrozole-resistant breast cancer with no *ESR1* mutations, reduced levels of ERα, and overexpression of HER2, lasofoxifene alone or combined with palbociclib inhibited primary tumor growth more effectively than fulvestrant. Lasofoxifene plus palbociclib also reduced bone metastases. These results suggest that lasofoxifene alone or combined with a CDK4/6 inhibitor may offer benefits to patients who have ER-low and HER2-positive, AI-resistant breast cancer, independent of *ESR1* mutations.

**Supplementary Information:**

The online version contains supplementary material available at 10.1186/s13058-024-01843-4.

## Introduction

Breast cancer is the most commonly diagnosed cancer and the leading cause of cancer mortality in women worldwide [1]. The estrogen receptor alpha (ERα) plays a key role in the progression of ER + breast tumors, which account for approximately 80% of all breast cancers [2]. Patients with ER + breast cancer are typically treated with endocrine therapy (ET) that antagonizes ER function (i.e., tamoxifen, fulvestrant [FUL]) or reduces estrogen production (i.e., aromatase inhibitors [AIs]) [3].

Aromatase inhibitors indirectly inhibit ER + tumor progression via estrogen deprivation [4]. While AIs surpass tamoxifen as first-line therapy for postmenopausal women with advanced breast cancer [5, 6], AI resistance develops in some patients [4, 7], often driven by activating mutations in the ligand binding domain of the ERα-encoding gene (*ESR1*) in up to 40% of AI-treated, metastatic breast cancer (mBC) [8–12]. Patients with advanced breast cancers resistant to AIs or ET can derive clinical benefit from FUL, a selective ER degrader (SERD), alone or combined with a CDK4/6 inhibitor (CDK4/6i) [13–18]. FUL with or without a CDK4/6i is the standard-of-care, second-line therapy for patients with advanced breast cancers after progression on AIs [19–21]. However, the utility of FUL is hindered by its poor bioavailability and pharmacokinetics [22, 23].

Lasofoxifene (LAS) is an oral, tissue-selective ER modulator that antagonizes ER function in breast cancer cells [24]. In the Postmenopausal Evaluation and Risk Reduction with Lasofoxifene (PEARL) trial, LAS reduced the incidence of ER + breast cancer in postmenopausal women with osteoporosis by 83% [25]. In preclinical studies, LAS alone or plus palbociclib (PAL) reduced tumor progression and metastases to a greater extent than FUL in a xenograft model of mBC harboring *ESR1* mutations [26]. Evidence of antitumor activity of LAS monotherapy or combined with abemaciclib has been demonstrated in the phase 2, ELAINE 1 and ELAINE 2 studies, respectively. In patients with ET-resistant, *ESR1*-mutated mBC who had prior CDK4/6i exposure, LAS monotherapy led to numerically longer progression-free survival than FUL (5.6 vs. 3.7 months; *P* = 0.138) [27], and LAS plus abemaciclib was associated with a median progression-free survival of approximately 13 months [28]. These promising results suggest that LAS may be an effective treatment for AI-resistant tumors.

To determine if LAS might be effective in AI-resistant breast cancers without *ESR1* mutations, we used an established, letrozole-resistant MCF7 LTLT breast tumor model that is not associated with an ERα-activating mutation [29, 30]. The antitumor activity of LAS alone or with PAL was compared with FUL or FUL plus PAL in this letrozole-resistant model.

## Materials and methods

### Cell culture

MCF7 LTLT cells (also known as LTLT-Ca cells [29, 30]) were a kind gift from Dr. Ganesh Raj at The University of Texas Southwestern Medical Center, and were originally derived from long-term, letrozole-treated MCF7 cells that were stably transfected with the aromatase gene (MCF7aro cells) [29, 30]. Cells were transfected with the L2G lentivirus vector (pFU-Luc2-eGFP) encoding luciferase and GFP under the control of a ubiquitin promotor [31] at a multiplicity of infection of 5 in suspension, and grown in RPMI containing 10% FBS and 1 µM letrozole.

### Genomic sequencing

Genomic DNA quality for MCF7 LTLT cells was assessed using Life Technologies Qubit quantification and Agilent TapeStation analysis. Whole genome sequencing (30X coverage) was completed by the University of Illinois at Chicago Genome Research Core on the Illumina NextSeq500 platform. Reads were aligned to the hg38 reference genome using Burrows-Wheeler Aligner (BWA-MEM) with removal of PCR duplicates using Picard. Variants were called using FreeBayes, and results were annotated using ANNOVAR.

### Western blot analysis

Cells were lysed in M-PER lysis buffer (Thermo fisher, 78,501) in the presence of protease inhibitor cocktail 3 (Calbiochem, 535,140). Samples were loaded on a WES Protein Simple platform. Antibodies against ERα (Santa Cruz, F10 sc8002), glucocorticoid receptor (GR; Cell signaling, 12041s), HER2 (Cell Signaling, 2242s), androgen receptor (AR; Sigma, sp242), or progesterone receptor (PR; KD68, an in-house generated rat monoclonal antibody [32]) were used.

### Breast cancer models

Mouse studies were performed in compliance with an approved Institutional Animal Care and Use Committee protocol at the University of Chicago. A mammary intraductal (MIND) mouse model that closely mimics the original ER + tumor [33, 34] was solely used, as it is a good representative, AI-resistant, mouse model for studying breast cancer tumor growth in the absence of *ESR1* mutations. NSG (NOD.Cg-Prkdc^scid^ Il2rg^tm1Wjl^/SzJ) mice (Jackson Laboratories, Bar Harbor, ME) were anesthetized via inhalation with 2–3% isoflurane in oxygen and injected with single-cell suspensions of GFP-luciferase-labeled MCF7 LTLT cells into mammary duct inguinal glands 4 and 9 (500,000 cells/gland), as previously described [26, 33, 34]. As per use in the MIND model, animals had intact ovaries (having estradiol levels close to that of a postmenopausal woman).

### Treatment

Mice were randomized to vehicle, LAS, FUL, PAL, LAS plus PAL, or FUL plus PAL (6 mice/group) 2–3 weeks after cell injections. Vehicle and LAS (10 mg/kg in 100 µL of PBS containing 15% PEG400) were administered subcutaneously 5 days/week, FUL (Med Chem Express, HY-13,636; 5 mg/mouse in 100 µL of mineral oil) subcutaneously once per week, and PAL (Med Chem Express, HY-50,567; 70 mg/kg in 100 µL of 50 mM sodium lactate buffer pH 4) via oral gavage 5 times/week. Clinically, LAS and FUL are administered orally and intramuscularly. However, for this study we injected both LAS and FUL subcutaneously for convenience; while that may be considered a limitation, equivalent biological responses with oral and subcutaneous administration of LAS and other SERMs were observed in preliminary, unpublished, preclinical dosing animal experiments. This experiment was repeated with the same design and 8–9 mice in each treatment group.

### Tumor growth measurement

Tumor growth in situ was monitored by imaging mice every other week in a Xenogen IVIS 200 instrument in the Integrated Small Animal Imaging Research Resource (University of Chicago). Tumors were assessed by imaging only because when cells are injected into the mammary duct in the MIND model, tumors form as a few smaller masses that follow the shape of the gland and are too small and soft to be palpable.

Prior to imaging, mice were injected with 100 µL of a 0.1 M luciferin solution in PBS (Perkin Elmer XenoLight, 122,799). The treatment groups were not blinded, as the same amount of luciferin was injected, and the mice were imaged on the same instrument after the same amount of time to help eliminate bias. At study end after 90–93 days of treatment (55–59 days of treatment in the repeat study), mice were injected with luciferin to measure luciferase activity and sacrificed 8 min later. Mammary gland tumors were excised and weighed. Liver, bones, brain, and lungs were removed and any metastases to these organs were measured by ex vivo imaging in the Xenogen IVIS 200 instrument.

### Histological analysis

Tissue was processed and histology analyzed by the Human Tissue Resource Center (HTRC; University of Chicago). Harvested tissues were fixed in formalin and sectioned for immunohistochemical (IHC) and hematoxylin and eosin (H&E) staining. Primary, excised glands were stained using antibodies against Ki67 (ThermoScientific, RM-9106-s, Clone SP6). H&E and IHC slides were scanned on a Nikon ECLIPSE Ti2 microscope with a 10X objective for high resolution images and analyzed with the NSI-Elements software. Tumor area was measured as a percentage of total tissue in the mammary gland based on H&E staining of representative sections. Proliferation of MCF7 LTLT primary tumors was determined by the percentage of Ki67-positive cells in total tumor cells using standardized manual counting.

### Statistical analysis

Graphs and boxplots were created using GraphPad Prism 9 software. *P*-values were determined using the nonparametric Kruskal Wallis test, with *P* < 0.5 considered statistically significant.

## Results

### Characterization of MCF LTLT cells via Western blot and genomic sequencing

**Fig. 1:**
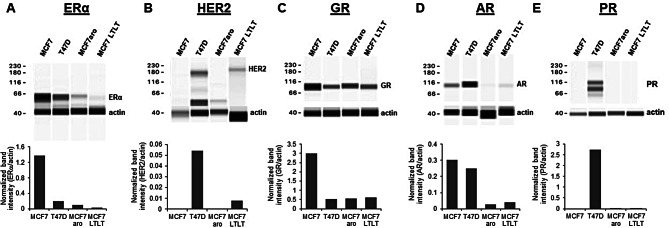
Protein expression profiles of MCF7 LTLT cells versus normal MCF7, T47D, and MCF7aro cells. The cells were assayed by immunoblot with antibodies against (**A**) ERα; (**B**) HER2; (**C**) GR; (**D**) AR; (**E**) PR. β-actin was used as an internal loading control. For AR and HER2, samples were run on a WES (ProteinSimple). For ERα, GR, and PR, one of two representative experiments were shown

Compared with normal MCF7 cells, MCF7 LTLT cells expressed lower levels of ERα, GR, and AR, but higher levels of HER2 (Fig. 1). The levels of ERα, AR, and PR in MCF7 LTLT cells were also lower than those in T47D cells, while the level of GR was similar between the two (Fig. 1).

Whole genome sequencing of MCF7 LTLT cells revealed a number of genetic variants compared with two MCF7 reference genomes (SRA accession numbers SRX7658479 and SRX513539). However, no ERα mutations were detected (Additional file 1: Fig. S1; Additional file 2: Table S1).

### Tumor growth

**Fig. 2:**
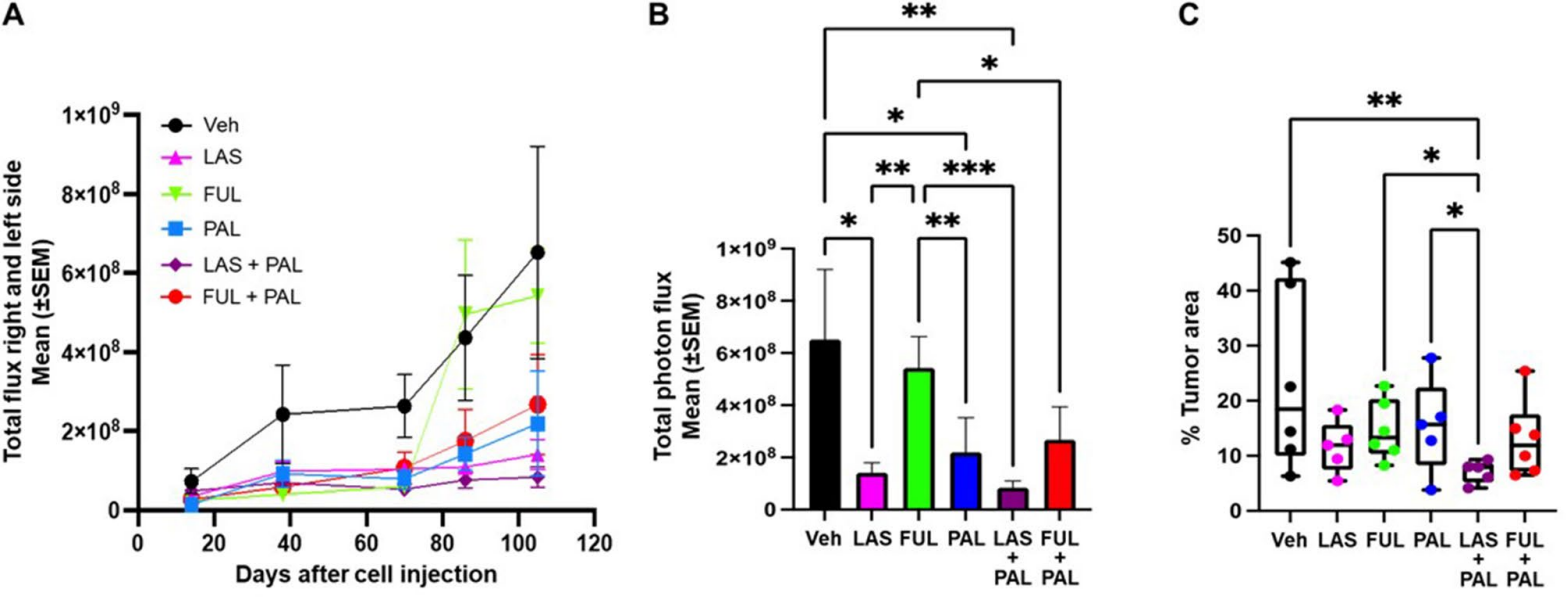
Progression of primary tumors in the LTLT breast cancer model (*n* = 5–6 mice). (**A**) Tumor growth over time via quantification of total photon flux from in vivo luminescence images. (**B**) Total photon flux of tumors at day 104 (end of study). (**C**) Box plot of percent tumor area measured by quantitative analysis of H&E staining over the gland area. Center line, median value; box, the 25th to 75th percentiles; whisker marks, the 5th and 95th percentiles. *P < 0.05, **P < 0.01, ***P < 0.001 by nonparametric Kruskal Wallis test. FUL, fulvestrant; LAS, lasofoxifene; PAL, palbociclib; SEM, standard error of the mean; Veh, vehicle

Tumors were not palpable in this mouse model. In vivo luminescence imaging showed that LAS, PAL, LAS/PAL, and FUL/PAL reduced tumor growth over time relative to vehicle and FUL (Fig. 2A). Total photon flux of tumors at study end (104 days after cell injection) was highest with vehicle versus any other treatment; signals with LAS and PAL alone were 4.6 and 3.0 times lower respectively, and that with LAS/PAL were 7.7 times lower, compared with vehicle (*P* < 0.05 vs. vehicle for all three groups; Fig. 2B). Total photon flux with LAS, PAL, LAS/PAL, and FUL/PAL was also significantly lower than with FUL (Fig. 2B). Percent tumor area in excised mammary glands was significantly lower for LAS/PAL versus vehicle and numerically lower with all other treatments versus vehicle (Fig. 2C; Additional file: Fig. S2). LAS/PAL was also associated with a significantly lower percent tumor area compared with FUL or PAL alone, but the difference between LAS/PAL and FUL/PAL groups was not statistically significant (Fig. 2C).

Similar results were seen in the repeat study, which showed better activity of LAS or LAS/PAL in reducing tumor burden compared with FUL alone as assessed by the total photon flux of tumors and percent tumor area (Additional file: Fig. S3).

### Tumor cell proliferation

**Fig. 3:**
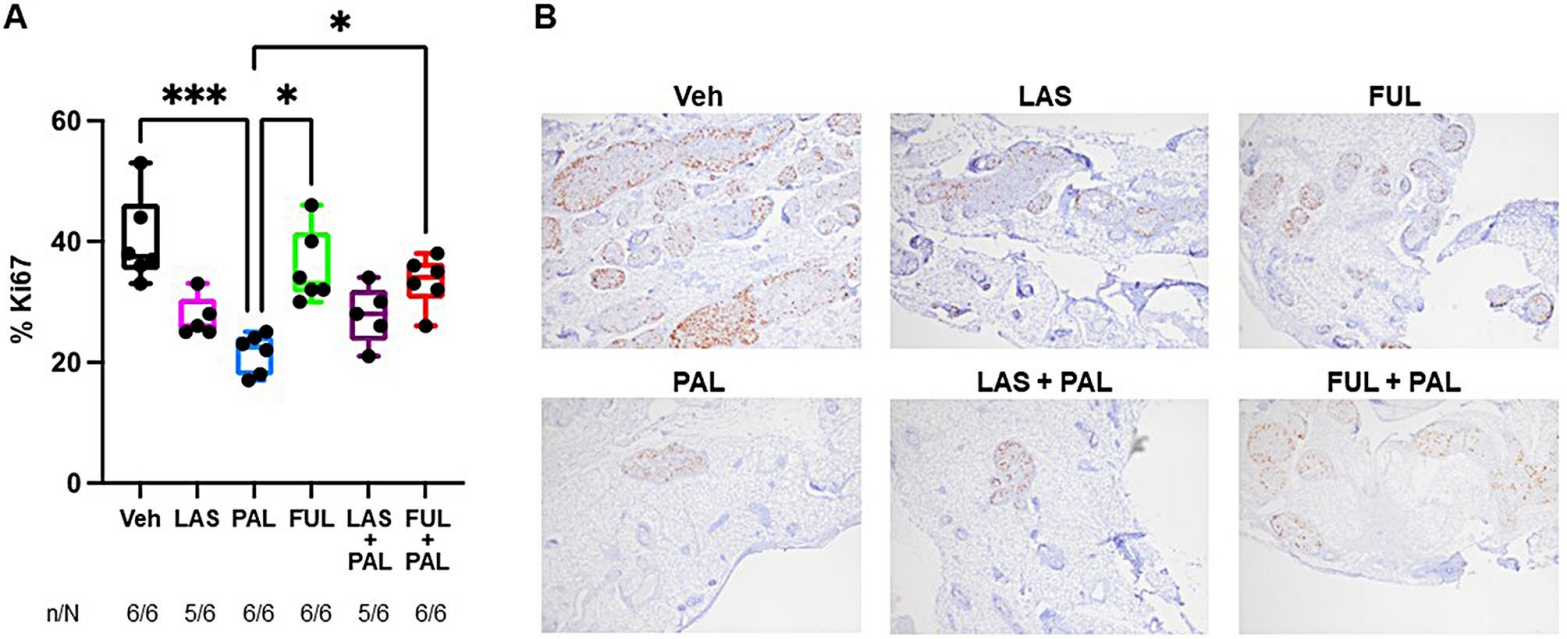
Tumor cell proliferation assessed by IHC staining for Ki67. (**A**) Box plot of Ki67% in the mammary gland determined. Center line, median value; box, the 25th to 75th percentiles; whisker marks, the 5th and 95th percentiles. n indicates the number of mice with Ki67 staining and N indicates the total number of mice at the end point of the study. (**B**) Representative IHC sections of Ki67 staining for each treatment group. *P < 0.05, **P < 0.01, ***P < 0.001 by nonparametric Kruskal Wallis test. FUL, fulvestrant; LAS, lasofoxifene; PAL, palbociclib; Veh, vehicle

Tumor cell proliferation indicated by Ki67 staining (mean ± SD) was numerically lower than vehicle (40.2%±7.3%) with LAS (27.4%±3.4%), FUL (35.7%±6.1%), and PAL (21.5%±3.3%), with the greatest difference, which reached statistical significance, between PAL and vehicle. Other statistically significant between-group differences were observed for PAL versus FUL and PAL versus FUL/PAL with PAL being most effective in reducing Ki67 (Fig. 3). The addition of PAL to LAS or FUL did not further reduce the Ki67% compared with each single agent (mean ± SD: 27.8%±4.8% with LAS/PAL and 33.3%±4.2% with FUL/PAL; Fig. 3).

These inhibitory effects on tumor proliferation were confirmed in the replicate study (Additional file: Fig. S4). The overall tumor cell proliferation was significantly reduced with LAS, LAS/PAL, and FUL/PAL compared with vehicle. The addition of PAL further reduced the Ki67 staining for FUL (*P* < 0.001 FUV vs. FUL/PAL), but not LAS. Additionally, only 1 of the 8 mice in the LAS/PAL group showed detectable Ki67 staining, while 6 of 8 mice in the LAS group and all mice in the PAL (8/8) and FUL/PAL (8/8) groups showed positive staining for Ki67.

### Tumor metastases

**Fig. 4:**
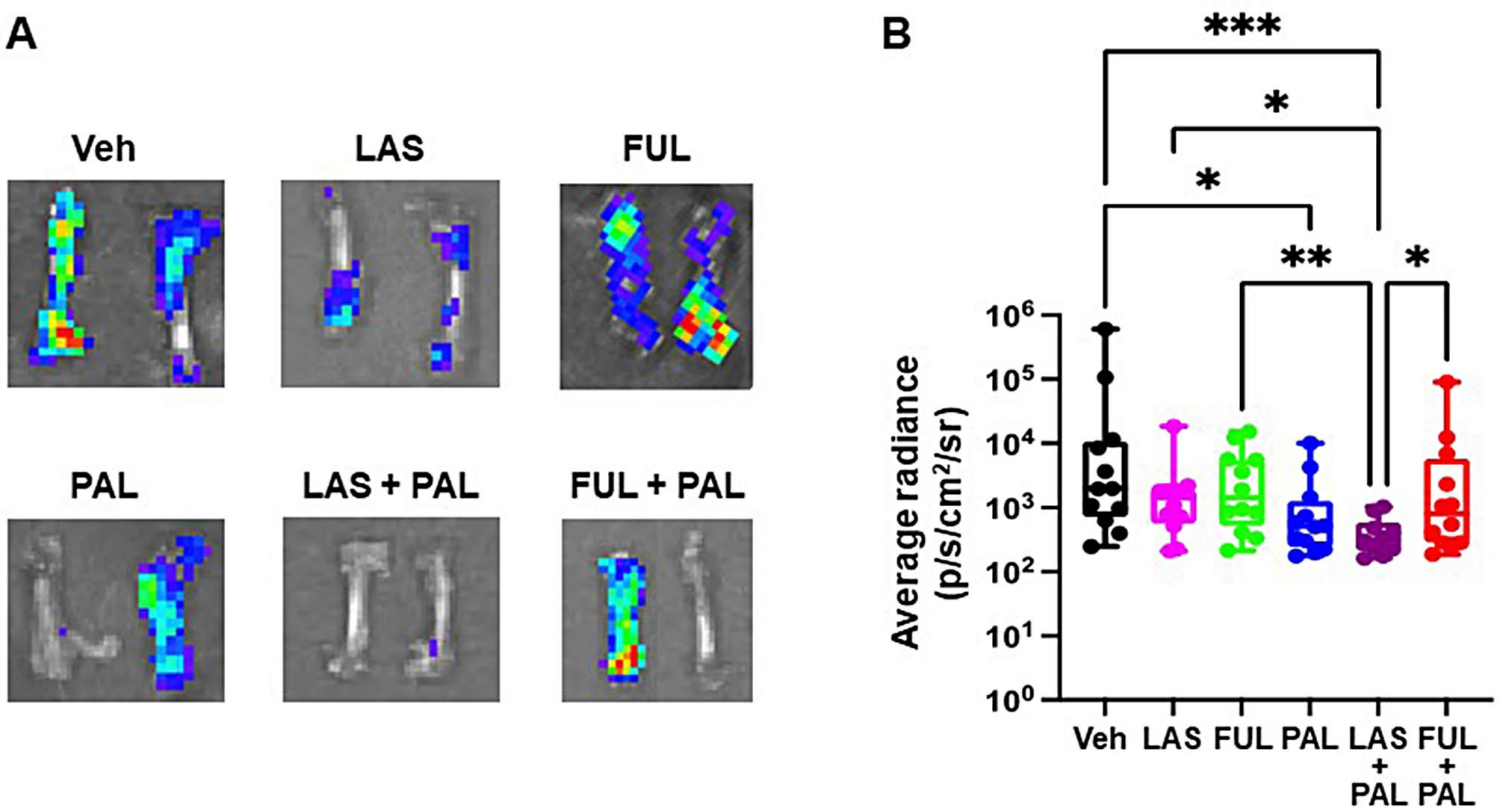
Bone metastases assessed by ex vivo luminescence imaging (*n* = 10–12 bones). (**A**) Representative ex vivo images of excised bones for each treatment group. (**B**) Box plot of ex vivo average radiance of the luciferin signals in the bones. Center line, median value; box, the 25th to 75th percentiles; whisker marks, the 5th and 95th percentiles. *P < 0.05, **P < 0.01, ***P < 0.001 by nonparametric Kruskal Wallis test. FUL, fulvestrant; LAS, lasofoxifene; PAL, palbociclib; Veh, vehicle

**Fig. 5:**
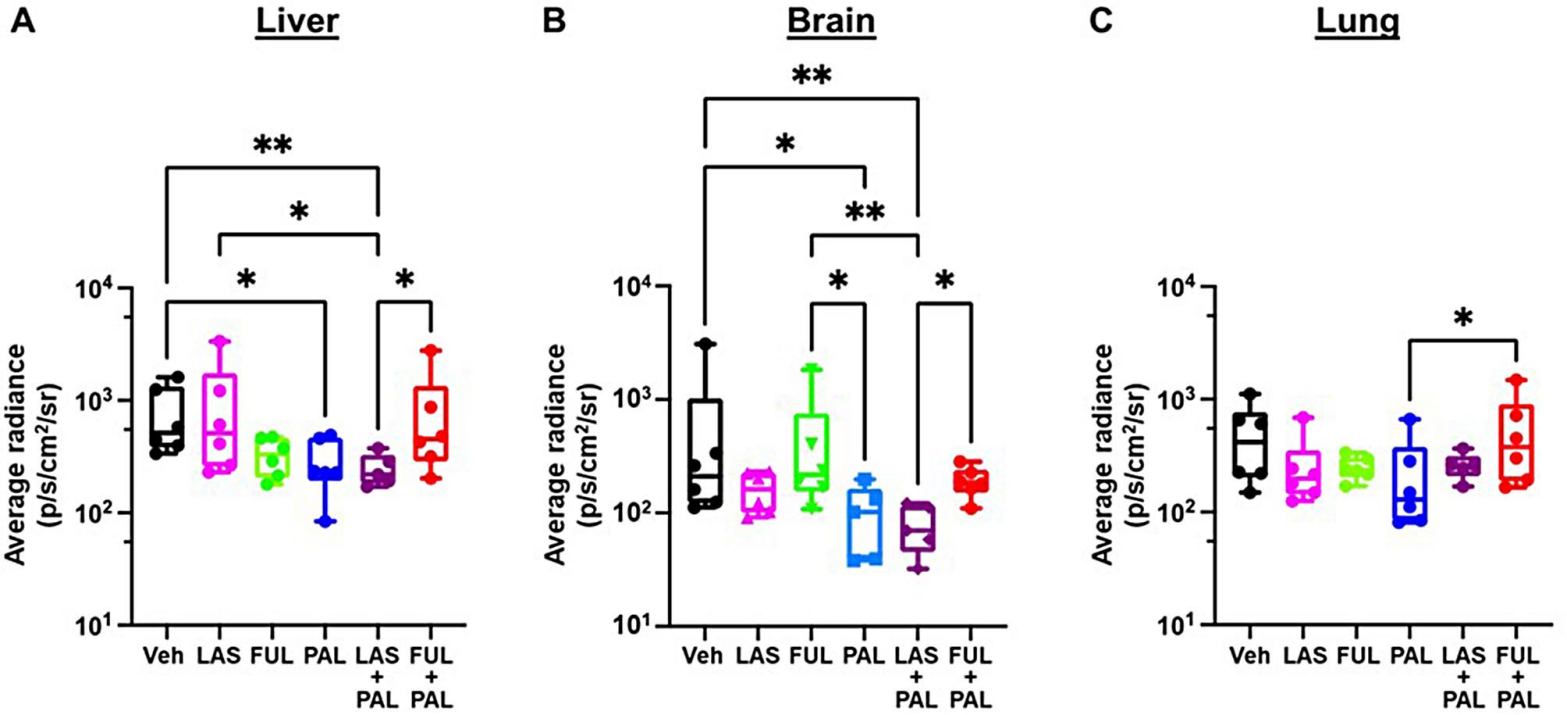
Metastases to distal sites assessed by ex vivo luminescence imaging (*n* = 5–6 mice). Box plot of ex vivo average radiance measured in excised (**A**) livers, (**B**) brains, and (**C**) lungs for each treatment group. Center line, median value; box, the 25th to 75th percentiles; whisker marks, the 5th and 95th percentiles. *P < 0.05, **P < 0.01, ***P < 0.001 by nonparametric Kruskal Wallis test. FUL, fulvestrant; LAS, lasofoxifene; PAL, palbociclib; Veh, vehicle

Quantification of ex vivo radiance of excised organs showed generally low radiance, indicating minimal metastases to distal sites (Figs. 4 and 5). Possible metastases to bone were detected (Fig. 4A); 6 bones in the vehicle group, 6 in the FUL group, and 5 in the FUL/PAL group showed signals above background threshold, but only 1 bone in the LAS/PAL group showed signal above background. Both PAL alone and LAS/PAL significantly reduced bone metastases relative to vehicle (Fig. 4B). LAS/PAL was also associated with significantly lower bone metastases compared with LAS, FUL, and FUL/PAL (Fig. 4B).

PAL and LAS/PAL consistently reduced metastases to the liver and the brain (*P* < 0.05 vs. vehicle), while other treatments did not provide a significant effect (Fig. 5A and B). LAS/PAL also resulted in significantly fewer liver metastases compared with LAS or FUL/PAL and fewer brain metastases compared with FUL or FUL/PAL (Fig. 5A and B). No statistically significant pattern for lung metastases was observed, except that the signal with PAL was significantly lower compared with FUL/PAL (Fig. 5C).

Trends of treatment effects on tumor metastases were generally similar in the repeat study (Additional file: Fig. S5).

## Discussion

In this study, the breast ER antagonist LAS, alone or combined with PAL inhibited primary tumor growth in a mouse model of AI-resistant breast cancer without *ESR1* mutations. Tumor cell proliferation was reduced in mice treated with LAS or LAS/PAL compared with other treatments. Bone metastases also decreased with LAS/PAL. Overall, LAS/PAL had the most anti-tumor activity versus vehicle in this model.

LAS, as an antiestrogen, is believed to exert its antitumor effects via breast tumor intrinsic ER antagonism [26]. Based on previously published data [29, 35] showing that neither tamoxifen nor FUL alone were effective second-line therapies for AI-resistant MCF7 tumor explants, it was not anticipated that LAS would inhibit the growth of LTLT breast tumors, which have very low ERα expression levels. However, LAS alone or combined with PAL inhibited tumor growth and exhibited significantly better antitumor activity compared with FUL or FUL/PAL in this LTLT model. While it is unclear why LAS, but not tamoxifen or FUL, is effective in this model, these data suggest that there is an imperfect correlation between ER levels and drug efficacy for different SERMs/SERDs. Certainly, the differences in efficacy of these molecules in the MIND model used here is not due to differences in receptor affinity. What we do know is when any one of these SERMs binds to the ER, a different conformation is invoked which will differentially impact transcriptional efficiency on a gene-by-gene basis for each SERM. Perhaps, the readout is more about receptor activation/inactivation versus “intrinsic antagonism.” Future mechanistic studies are needed to address the unexpected response difference between LAS and FUL.

The different doses of LAS and FUL could be a possible reason for their different activities in our experiments. However, based on several previous, unpublished results in preliminary animal studies evaluating optimal dosing and administrative routes for LAS and FUL, we know we used doses that saturate the ER and produce biological responses related to inhibiting breast cancer growth. While it is difficult to extrapolate such preclinical doses in mice and rats to humans, we know that the clinically effective doses of LAS and FUL are different and are delivered differently (oral vs. intramuscular). Further, recently published clinical data from the ELAINE 2 study showed that LAS had greater anti-tumor activity than FUL when treating patients with metastatic breast cancer harboring *ESR1* mutations [28].

Of interest, fewer mice in the LAS and LAS/PAL groups showed detectable signals with Ki67 staining, especially in the repeat experiment which had a shorter treatment duration before tissue collection. In addition, the Ki67% was significantly lower with LAS or LAS/PAL compared with FUL or vehicle in the repeat study. These tumor proliferation results suggest that LAS alone or the LAS/PAL combination may delay the onset of cell proliferation. Although tumors were less progressive, as expected for a MIND model, which preserves the luminal phenotype of the original ER + tumors [34], and bone metastasis levels were low, a greater reduction in bone metastases with LAS/PAL versus vehicle, FUL, or FUL/PAL was clear. These results suggest that relatively high ER expression might not be required for LAS to have antitumor effects. Thus, LAS and LAS/PAL could be potentially useful for treating ER-low breast cancers, which have been shown to lack response to ET [36]. In fact, one can easily hypothesize that the effect of LAS is not solely an ERα-mediated event.

In the current study, FUL did not elicit clear antitumor activity in the LTLT xenografts, which is in agreement with earlier findings that the ER-independent LTLT cells were insensitive to FUL [29, 30]. As in vitro evaluations of LTLT cells were not conducted in our study, we cannot exclude the possibility that FUL may be active in cell models but less so in vivo due to its poor pharmacokinetics [22, 23]. The low activity of FUL and FUL/PAL compared with LAS or LAS/PAL may also be attributable to poor drug availability and an inability to saturate tumor ERs with FUL [22, 23, 37]. While the exact pharmacokinetics and bioavailability of LAS and FUL are not known in the MIND model, prior dose-finding studies have shown anti-tumor effects in breast cancer models at the LAS and FUL doses used.

Upregulation of HER2 has been found in several AI-resistant breast cancer cell lines, suggesting the involvement of growth factor signaling pathway activation in AI resistance [38, 39]. When treating HER2 + breast cancers, addition of a HER2-targeted therapy is common [19]. In LTLT breast cancer xenograft models, downregulating HER2/mitogen-activated protein kinase (MAPK) pathway was shown to restore the sensitivity of tumors to letrozole [29, 30]. LAS either alone or combined with a CDK4/6i was shown to inhibit the growth of ER+/HER2- tumors with mutant ERs in preclinical and clinical studies [26–28], but its activity has not been tested in HER2 + breast cancers. Results from the current study suggest the potential of LAS and LAS/PAL for inhibiting HER2-overexpressing breast tumors. However, additional studies are needed to confirm the efficacy of LAS on HER2 + tumors. Whether LAS has any effects on blocking growth factor signaling pathways also warrants further investigation.

Some aspects of our experimental design may limit our studies. The different doses of LAS and FUL could be a possible reason for their different activities in our experiments. However, based on several previous, unpublished results in preliminary animal studies evaluating optimal dosing and administrative routes for LAS and FUL, we know we used doses that saturate the ER and produce biological responses related to inhibiting breast cancer growth. While it is difficult to extrapolate such preclinical doses in mice and rats to humans, we know that the clinically effective doses of LAS and FUL are different and are delivered differently (oral vs. intramuscular). Further, recently published clinical data from the ELAINE 2 study showed that LAS had greater anti-tumor activity that FUL when treating patients with metastatic breast cancer harboring *ESR1* mutations [28]. The subcutaneous administration of LAS in our experiments when LAS is administered orally in women, may also be a limitation, despite our previous, unpublished observations of equivalent responses with oral and subcutaneous administration of LAS and other SERMs in animal models.

Our results may also be limited by the use of one animal model, although the intraductal tumors of the MIND mouse model closely mimic original ER + tumors [33, 34]. In addition, the model is a good representative, AI-resistant, mouse model for studying breast cancer tumor growth in the absence of *ESR1* mutations, and no other equivalent model is available. Further, the MIND model uses an intact animal and it is unknown whether its endogenous estrogens would interfere with our reported effects of LAS and FUL. However, estradiol is required for MCF7 breast tumor growth, and the given SERM/SERDs are known to block the effects of estradiol.

## Conclusions

In a model of AI-resistant breast cancer without *ESR1* mutations, LAS alone or combined with PAL inhibited the growth of primary tumors more effectively than FUL. In addition, the LAS/PAL combination significantly reduced bone metastases. These results suggest that LAS alone or in combination with a CDK4/6i may be a promising therapy for patients with AI-resistant breast cancer, independent of *ESR1* mutations. These results also suggest that LAS might be effective in tumors that express low levels of ERα.

### Electronic supplementary material

Below is the link to the electronic supplementary material.

Supplementary Material 1

Supplementary Material 2

Supplementary Material 3

Supplementary Material 4

Supplementary Material 5

Supplementary Material 6

Supplementary Material 7

Supplementary Material 8

## Data Availability

All data generated or analyzed during the current study are available from the corresponding author on reasonable request. The sequencing data generated and analyzed during the current study are available through SRA (accession number SRX21863507).
