# Supplementary figures and images for: Lasofoxifene as a potential treatment for aromatase inhibitor-resistant ER-positive breast cancer
Source: Breast Cancer Res. 2024 Jun 7;26:95. doi: 10.1186/s13058-024-01843-4 (PMC11161925; doi:10.1186/s13058-024-01843-4)

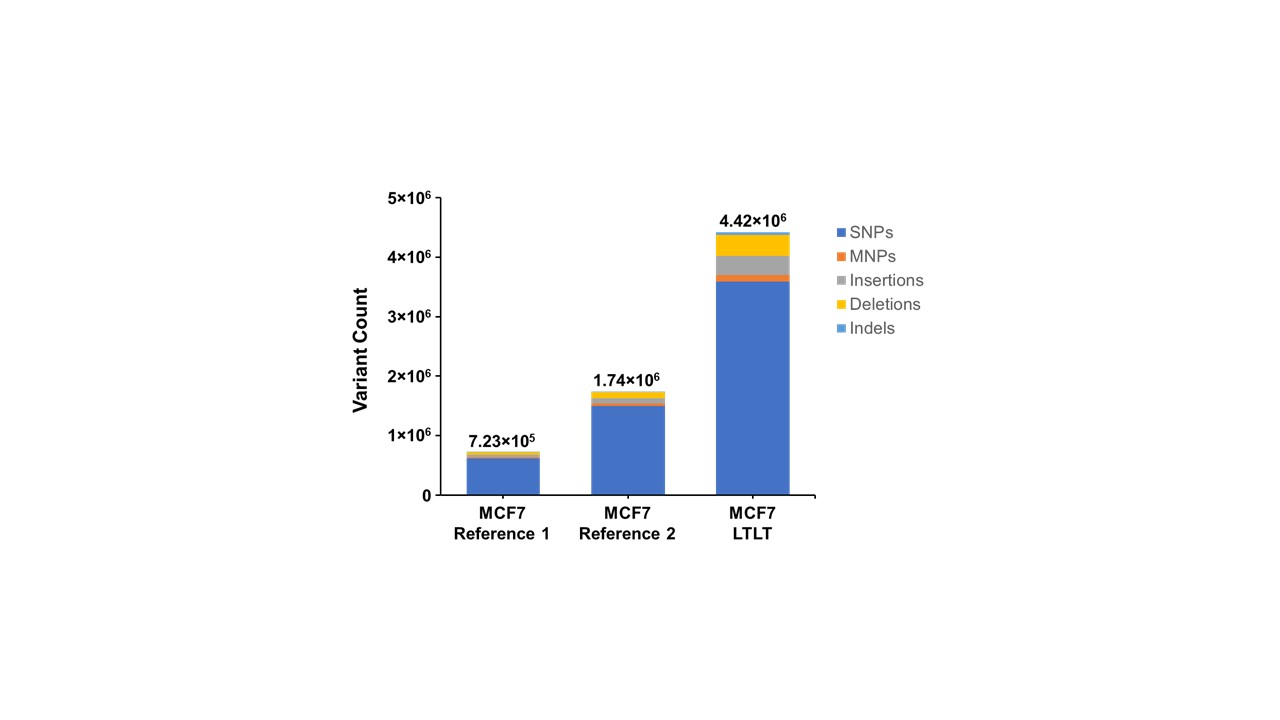

Supplement: Supplementary file 1 — Supplementary Material 1 [file 13058_2024_1843_MOESM1_ESM.jpg]

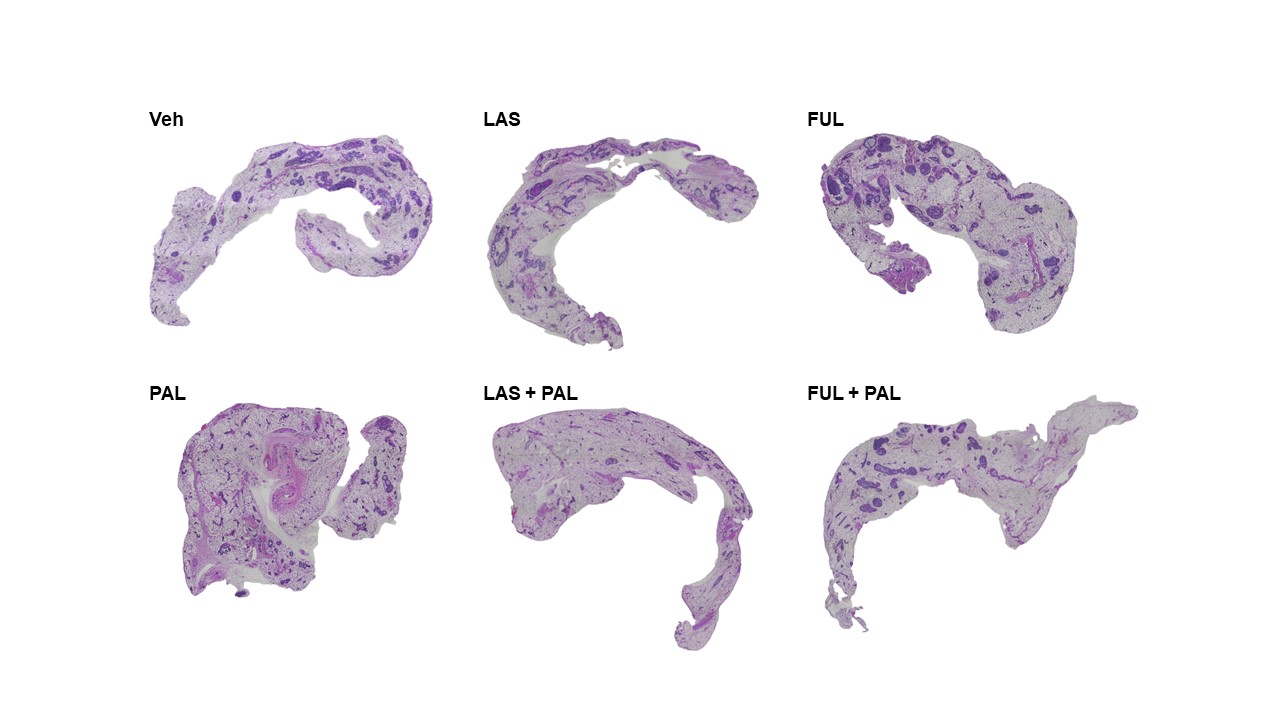

Supplement: Supplementary file 2 — Supplementary Material 2 [file 13058_2024_1843_MOESM2_ESM.jpg]

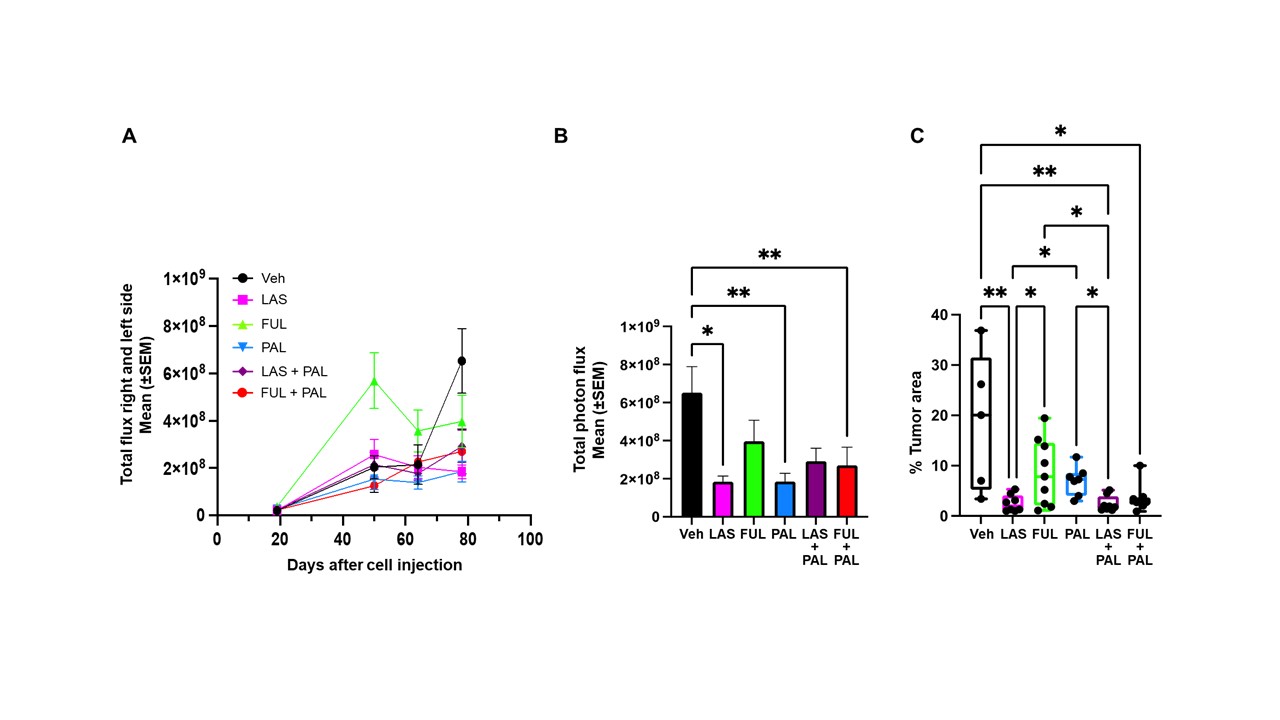

Supplement: Supplementary file 3 — Supplementary Material 3 [file 13058_2024_1843_MOESM3_ESM.jpg]

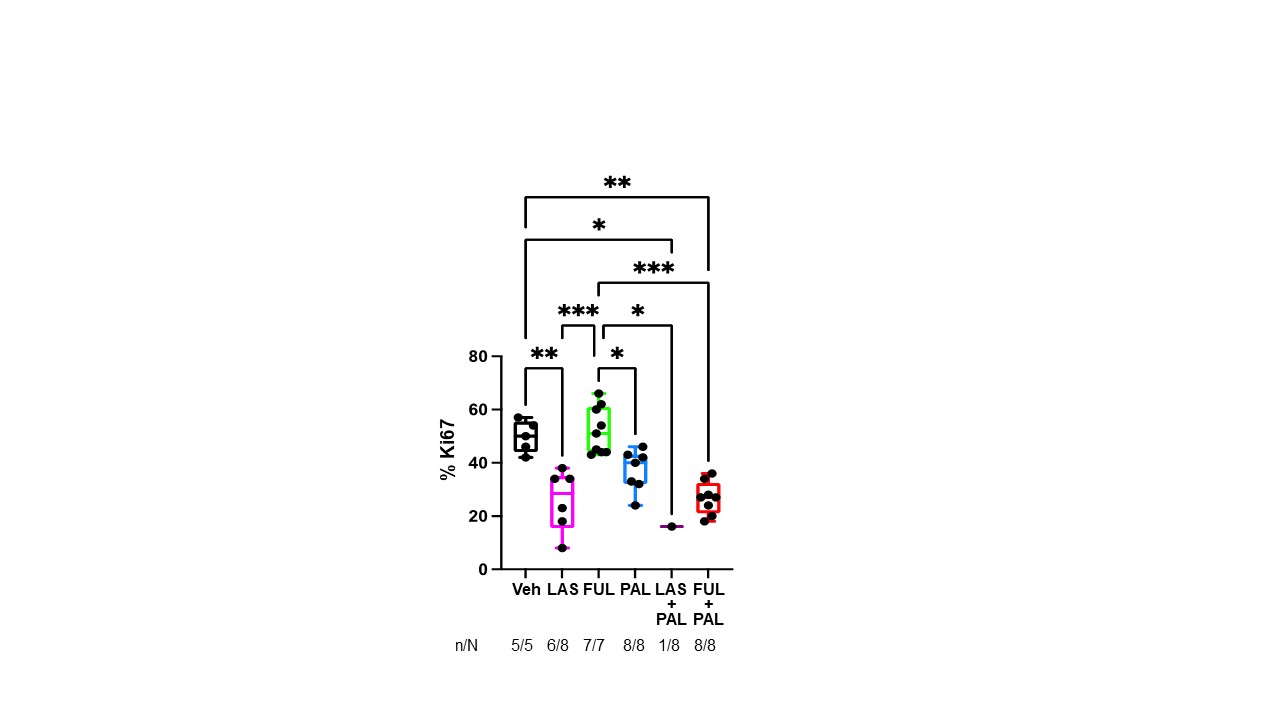

Supplement: Supplementary file 4 — Supplementary Material 4 [file 13058_2024_1843_MOESM4_ESM.jpg]

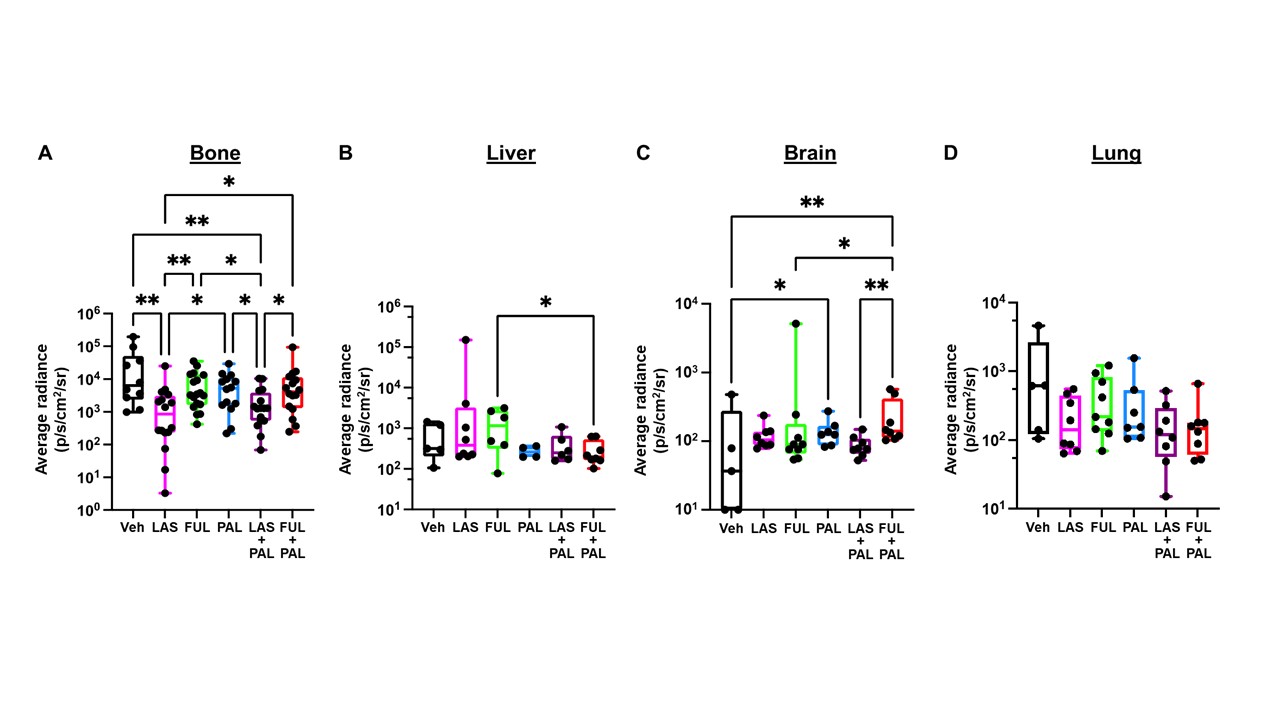

Supplement: Supplementary file 5 — Supplementary Material 5 [file 13058_2024_1843_MOESM5_ESM.jpg]
