# Supplementary material for: Lasofoxifene as a potential treatment for aromatase inhibitor-resistant ER-positive breast cancer
Source: Breast Cancer Res. 2024 Jun 7;26:95. doi: 10.1186/s13058-024-01843-4 (PMC11161925; doi:10.1186/s13058-024-01843-4)
Supplement: Supplementary file 6 — Supplementary Material 6 [file 13058_2024_1843_MOESM6_ESM.docx]

# Additional supplemental figures

**Fig. S1** Number of genomic variants identified in MCF7 reference 1 (SRX7658479), MCF7 reference 2 (SRX513539), and MCF7 LTLT cells. Variants are characterized as SNPs, MNPs, insertions, deletions, or indels. Total variant counts are indicated above each stacked bar. Indel, insertion-deletion; MNP, multiple nucleotide polymorphism; SNP, single nucleotide polymorphism.

**Fig. S2** H&E staining of one representative mammary gland for each treatment group.

**Fig. S3** Progression of primary tumors in the LTLT breast cancer model in Study 2 (n=5–9 mice). (**A**) Tumor growth over time via quantification of total photon flux from in vivo luminescence images. (**B**) Total photon flux of tumors at day 78 (end of study). (**C**) Box plot of percent tumor area measured by quantitative analysis of H&E staining over the gland area. Center line, median value; box, the 25th to 75th percentiles; whisker marks, the 5th and 95th percentiles. **P*<0.05, ***P*<0.01, ****P*<0.001 (by nonparametric Kruskal Wallis. FUL, fulvestrant; LAS, lasofoxifene; PAL, palbociclib; Veh, vehicle.

**Fig. S4** Box plot of Ki67 percentage in the mammary gland determined in Study 2. Center line, median value; box, the 25th to 75th percentiles; whisker marks, the 5th and 95th percentiles. **P*<0.05, ***P*<0.01, ****P*<0.001 by nonparametric Kruskal Wallis. n indicates the number of mice with Ki67 staining and N indicates the total number of mice at the end of the study. FUL, fulvestrant; LAS, lasofoxifene; PAL, palbociclib; Veh, vehicle.

**Fig. S5** Metastases to distal sites assessed by ex vivo luminescence imaging in Study 2 (n=5–9 mice). Box plot of ex vivo average radiance measured in excised (**A**) bones, (**B**) livers, (**C**) brains, and (**D**) lungs for each treatment group. Center line, median value; box, the 25th to 75th percentiles; whisker marks, the 5th and 95th percentiles. **P*<0.05, ***P*<0.01, ****P*<0.001 by nonparametric Kruskal-Wallis. FUL, fulvestrant; LAS, lasofoxifene; PAL, palbociclib; Veh, vehicle.
