# Supplementary material for: Lasofoxifene as a potential treatment for aromatase inhibitor-resistant ER-positive breast cancer
Source: Breast Cancer Res. 2024 Jun 7;26:95. doi: 10.1186/s13058-024-01843-4 (PMC11161925; doi:10.1186/s13058-024-01843-4)
Supplement: Supplementary file 8 — Supplementary Material 8 [file 13058_2024_1843_MOESM8_ESM.docx]

# Additional supplemental table

**Table S1.** Novel variants identified in MCF7 LTLT cells, compared with two MCF7 reference datasets (SRA accession numbers SRX7658479 and SRX513539). No variants in *ESR1* were identified. Sequencing data are available through SRA accession number SRX21863507.
